# Breaking Through the Bottleneck of Wireless Physical-Layer Key Generation by Dynamic Agile Reconfigurable Intelligent Surface Antenna (DARISA)

**DOI:** 10.3390/e28020146

**Published:** 2026-01-28

**Authors:** Yonglin Ma, Hui-Ming Wang

**Affiliations:** School of Information and Communications Engineering, Xi’an Jiaotong University, Xi’an 710049, China; mayonglin@stu.xjtu.edu.cn

**Keywords:** intelligent metasurface antenna, physical-layer key generation, quasi-static channel, key capacity

## Abstract

In widely deployed Internet of Things (IoT) scenarios, physical-layer key generation (PLKG) serves as a useful complement to conventional cryptographic methods, yet it often suffers from a fundamentally low key generation rate, which becomes particularly severe in quasi-static environments. This low rate is mainly attributed to three key issues: (1) slow channel variations, which provide insufficient randomness and thus limit the key generation rate; (2) correlation between the legitimate channel and the eavesdropping channel, which reduces the uniqueness of the extracted key and further degrades the achievable rate; and (3) insufficient degrees of freedom in the key source, which constrain the key space. To address these challenges, this paper introduces the Dynamic Agile Reconfigurable Intelligent Surface Antenna into physical-layer key generation. By deploying metasurface antennas at both ends and independently applying random phase modulation, the scheme injects two-sided randomness, thereby mitigating the adverse effects of quasi-static channels and legitimate eavesdropper channel correlation. Moreover, by leveraging the dynamic, agile, and reconfigurable characteristics of the metasurface antennas in the key generation process, the proposed approach can further enhance the key generation rate while simultaneously resolving all three issues above. The proposed scheme is developed under a general setting where correlation exists between the legitimate and eavesdropping channels. A closed-form expression for the key capacity is rigorously derived, accompanied by detailed theoretical analysis and simulations. The results demonstrate the superiority of the proposed approach when applied to physical-layer key generation.

## 1. Introduction

With the rapid evolution of wireless communication technologies, the Internet of Things (IoT) is imperceptibly reshaping people’s daily lives. In key application domains such as environmental monitoring, industrial control, and smart homes, IoT has not only penetrated deeply and played a fundamental role, but has also brought great convenience to everyday production and life [1]. Looking towards the future, two core requirements are becoming increasingly important: securely and reliably connecting massive IoT devices to the network, and establishing dependable secure communication links among multiple devices. However, traditional security solutions usually rely on high-complexity key generation mechanisms and encryption–decryption algorithms. Considering that IoT devices are typically limited in power consumption and computing capability, such complex operations are often unaffordable. Therefore, relying solely on conventional cryptographic algorithms is no longer sufficient to meet the practical security requirements of IoT scenarios [2].

Physical-layer key generation schemes exploit the short-term reciprocity, spatial decorrelation, and time-varying characteristics of wireless channels to efficiently generate symmetric keys for legitimate users, providing a lightweight security solution for secure IoT access and secure links among multiple devices [3]. Currently, physical-layer key generation has been widely studied and has been experimentally applied to WiFi, ZigBee, LoRa, and Bluetooth systems in [4,5,6,7]. Due to its low computational complexity and low communication overhead, this approach is particularly suitable for resource-constrained scenarios. For example, [8] applies physical-layer key generation to V2X scenarios, where high vehicle mobility makes key distribution and management challenging, while [9] points out that, for rapidly deployed and highly maneuverable unmanned aerial vehicle (UAV) scenarios, secure communication can be achieved by quickly establishing temporary keys over highly mobile and time-varying channels. In addition, physical-layer key generation has been introduced into satellite communications in [10] and into medical applications in [11], where keys are generated from wireless fading and multipath characteristics in short-range, body-centric environments to protect sensitive medical data.

The central problem in physical-layer key generation is how to enhance the key generation rate. The low key rate is mainly related to the following three key issues: (1) slow channel variation leads to an insufficient key generation rate, since physical-layer key generation relies on channel time variability and quasi-static channels provide limited randomness; (2) correlation between the legitimate and eavesdropping channels degrades the final secret key rate [12]; and (3) insufficient degrees of freedom (DoFs) of the key source restrict the key space, as the DoFs are often limited by the number of transmit and receive antennas, and an insufficient number of antenna DoFs inevitably constrains the achievable key rate.

Several studies have proposed methods to address the above three issues. For issue (1), Refs. [13,14] introduce artificial randomness, such as transmitting artificial random signals or deliberately perturbing the received signal, to enrich the randomness of the key source. For issue (3), Ref. [15] deploys multiple antennas or multiple relays to obtain multidimensional time–frequency–space channel resources, thereby enlarging the key space. In recent years, advances in new materials have also brought fresh ideas for tackling these challenges. Ref. [16] introduces reconfigurable intelligent surfaces (RIS), whose panels are composed of a large number of controllable reflecting elements based on novel electromagnetic materials and can flexibly manipulate the propagation of electromagnetic waves to create a programmable wireless environment. By deploying RIS as an intelligent reflecting surface (IRS) between the transmitter and receiver, the wireless environment can be effectively reshaped. In [17], randomly configuring the phase of IRS elements is shown to construct highly time-varying and random channels, which helps address issue (1). Furthermore, Ref. [18] designs a channel probing protocol that extracts randomness from finer-grained equivalent channels, thereby further improving the key generation rate. For issue (2), Ref. [19] proposes superimposing orthogonal artificial noise on the legitimate channel to interfere with the eavesdropper’s received signal, and [20] formulates an optimization problem to maximize the secret key rate when the legitimate and eavesdropping channels are correlated by jointly optimizing the IRS phase configuration. In addition, STAR-RIS-assisted physical-layer key generation has also been studied in [21].

Besides using intelligent surfaces purely as reflecting panels, another approach is to integrate electromagnetic metamaterials with radio-frequency (RF) front-end circuits to construct intelligent metasurface antennas with environment-reconfigurable capabilities. In this work, we consider a Dynamic Agile Reconfigurable Intelligent Surface Antenna (DARISA), which consists of a large number of reconfigurable programmable elements and connects each antenna to a single RF chain. DARISA can actively exchange channel state information and flexibly tune the electromagnetic response of each programmable element, thus reshaping the wireless environment. Owing to its dynamic agility, DARISA can rapidly adjust the metasurface phase response multiple times within one symbol duration, enabling multiple observations of the signal in different dimensions without increasing the signal bandwidth, as discussed in [22]. By introducing DARISA into physical-layer key generation and appropriately exploiting its capabilities, the three critical issues that limit the key rate can be addressed simultaneously. In quasi-static environments, DARISA can effectively enhance the randomness of the key source and reduce the correlation between the legitimate and eavesdropping channels. At the same time, its dynamic agile property can expand the key space, further improving the key generation rate.

Research on metasurface-antenna-assisted physical-layer key generation is still relatively limited. In [23], a key generation scheme based on received signals assisted by a Dynamic Metasurface Antenna (DMA) was proposed, while [24] proposes a scheme that deploys a single-end DMA to generate keys from equivalent channels. In the above DMA-assisted physical-layer key generation schemes, when correlation exists between the legitimate channel and the eavesdropping channel, key leakage may occur, which in turn degrades the achievable key generation rate. In this work, we deploy DARISA at both ends of the legitimate users and introduce two-sided randomness by applying independent random phase modulation at each side, thereby addressing the rate degradation caused by legitimate eavesdropper channel correlation. The DARISA adopted in this paper is fundamentally different from the DMA antennas mentioned above, featuring a more advanced structure and better performance. Compared to other physical layer key generation schemes, this approach does not introduce excessive redundant steps and resolves numerous challenges in the field of physical layer key generation through improvements made solely at the device level. Moreover, by leveraging DARISA’s inherent dynamic agility, superior performance can be achieved. Consequently, it exhibits strong potential for broad practical deployment. By exploiting its dynamic and agile reconfigurability in physical-layer key generation, the proposed approach can expand the key space and further improve the key generation rate.

- This paper proposes a channel-based key generation scheme for dual-end deployment of DARISA under quasi-static conditions, as well as an enhanced version incorporating dynamic agility features, specifically designed for scenarios where the legitimate channel and eavesdropping channel exhibit correlation.
- We provide detailed theoretical analysis of the proposed schemes and derive a closed-form expression for the key capacity, together with an in-depth study of the impact of the number of agile switching operations on performance.
- We conduct MATLAB (Version R2025b) simulations to evaluate the proposed schemes and compare them with existing DMA-assisted and RIS-assisted physical-layer key generation schemes. The results demonstrate that double-sided DARISA deployment, further combined with its dynamic agility, can effectively enhance the key generation rate and constitutes an innovative physical-layer key generation solution.

## 2. System Model

### 2.1. Structure of DARISA

**Figure 1:**
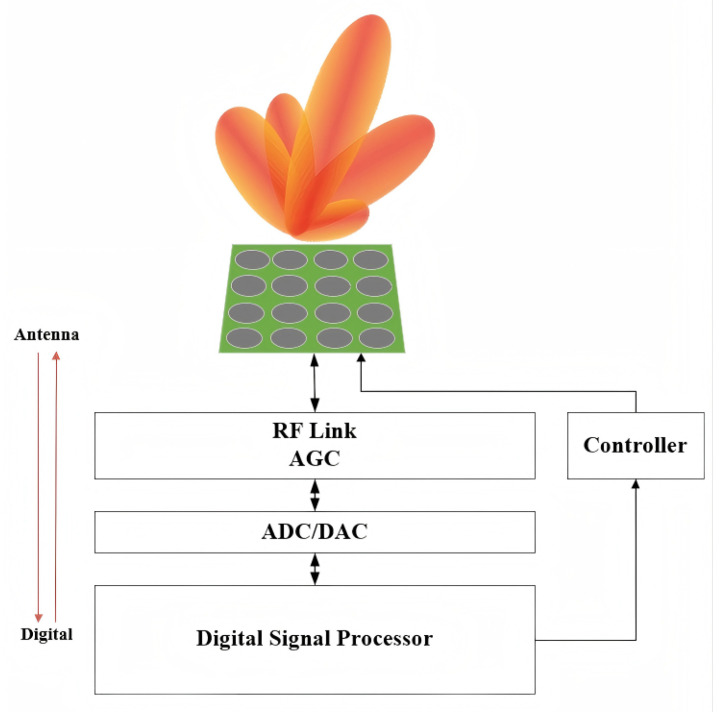
Structure of Dynamic Agile Reconfigurable Intelligent Surface Antenna.

The literature [22] first proposed DARISA and detailed its structural features and advantageous characteristics. Its hybrid analog-digital beamforming structure is shown in Figure 1. Unlike the conventional use of an intelligent metasurface as a passive reflector to form an intelligent reflecting surface (IRS), DARISA directly employs the intelligent metasurface as an antenna. By exploiting the sensing and controllable properties of the metasurface, DARISA overcomes the fixed, non-tunable electromagnetic response of traditional antennas after fabrication and thus acts as a new type of intelligent antenna. Compared with conventional smart antennas that rely solely on multi-channel digital-domain signal processing, DARISA provides flexible control and signal-processing capability in both the antenna domain and the digital domain, with the antenna-domain reconfigurability being particularly distinctive.

The system architecture of DARISA is shown in Figure 1. It consists of a power distribution network, amplitude–phase control circuits, and patch-type radiating antennas. The power distribution network splits the incident RF signal into equal-power branches and delivers them to each metasurface antenna element. The amplitude–phase control circuits modulate the magnitude and phase of the RF signals received by each element, while the patch radiators convert the modulated RF signals into free-space radiation. As a planar antenna, DARISA is composed of a large number of square metamaterial unit cells. Each unit cell integrates controllable electromagnetic components whose state can be adjusted to manipulate the amplitude, phase, and other parameters of the transmitted or received signals.

The dynamic agile property of the DARISA system is enabled by the parallel feeding architecture and the nanosecond-level response of the metasurface elements (for example, an implementation using PIN diodes can achieve a response time on the order of 2 ns [25]). In contrast to dynamic metasurface antennas (DMA) driven by a waveguide with sequentially activated elements, DARISA simultaneously excites all elements through a parallel coaxial feeding network. Owing to the response time being much shorter than the symbol period, multiple phase configurations can be realized within a single symbol duration. In practice, however, the number of agile reconfigurations is limited by the speed of the hardware switches and the performance of the control circuitry. The number of reconfigurations *K* cannot grow unbounded and must satisfy a power-splitting constraint: given a total transmit power *P*, if *K* reconfigurations are performed within one symbol period, the effective transmit power of each phase state is P/K. This normalization (time-sharing) model is used to ensure a fair comparison of different values of *K* under the fixed total energy budget per symbol. It does not imply a physical power splitter, but rather is introduced for theoretical analysis. In practice, the effective transmit power of each phase state is P/K within one symbol period, where *K* is limited by hardware reconfiguration delays and control circuit performance.

**Figure 2:**
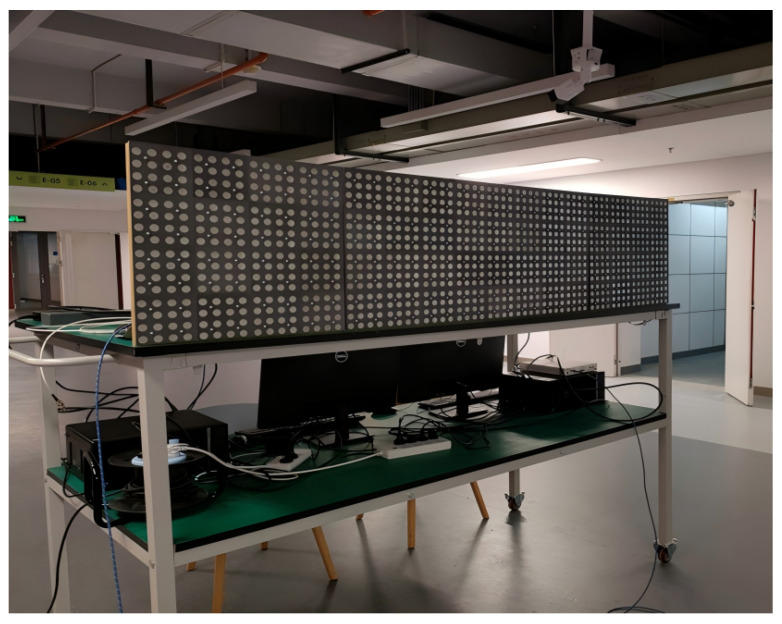
Prototype implementation of Dynamic Agile Reconfigurable Intelligent Surface Antenna.

By exploiting this dynamic agility, multiple observations of the signal in different dimensions can be obtained without increasing the signal bandwidth. In this work, the dynamic agile property of DARISA is used to enlarge the key space, thereby further improving the key generation rate. Our current physical prototype is shown in Figure 2. It achieves a switching time of approximately 20 ns. In IoT scenarios with typical 1 MHz bandwidth, the achievable agile switching rate K reaches 50. In the simulation experiments presented in the paper, K was demonstrated using more conservative values. As hardware advances, this approach will achieve superior performance.

### 2.2. Signal Transmission and Reception Model

**Figure 3:**
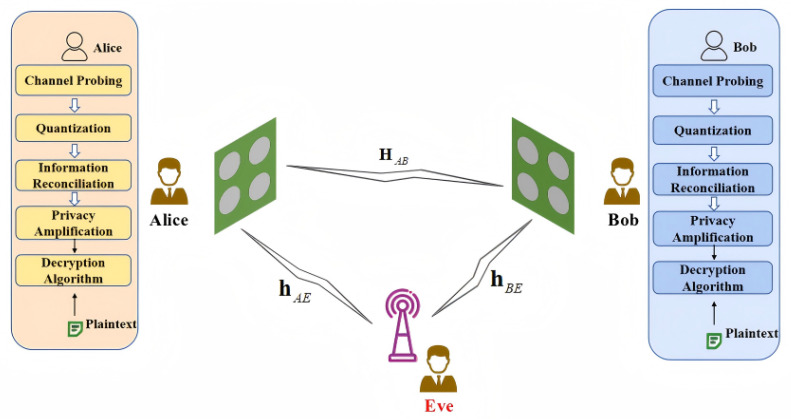
Dual-ended DARISA-based key generation system model.

The system model considered in this paper is shown in Figure 3. The wireless channels are modeled as Rayleigh fading channels. Alice and Bob are both equipped with an *N*-element DARISA, while Eve is equipped with a conventional single-antenna receiver. The configurable phase-shift vectors are denoted by $v_{A}={\left[e^{j\theta_{A,1}},e^{j\theta_{A,2}},\ldots ,e^{j\theta_{A,N}}\right]}^{H},v_{B}={\left[e^{j\theta_{B,1}},e^{j\theta_{B,2}},\ldots ,e^{j\theta_{B,N}}\right]}^{H}$, where $\theta_{A,i},\theta_{B,i}∼U\left(0,2\pi \right)$. The channel from Alice to Bob is denoted by $H_{AB}\in C^{N\times N}$, where the element in the *i*-th row and *j*-th column is $h_{AB,ij}$ with $h_{AB,ij}∼\mathrm{CN}\left(0,\sigma_{b}^{2}\right)$. The channels from Alice to Eve and from Bob to Eve are denoted by $h_{AE},h_{BE}\in C^{N\times 1}$, whose *i*-th elements are $h_{AE,i}$ and $h_{BE,i}$, respectively, with $h_{AE,i}∼\mathrm{CN}\left(0,\sigma_{ae}^{2}\right)$ and $h_{BE,i}∼\mathrm{CN}\left(0,\sigma_{be}^{2}\right)$. All noise terms mentioned in this paper, $n_{A}$, $n_{B}$, $n_{AE}$, and $n_{BE}$, are modeled as zero-mean complex Gaussian random variables with variance $\sigma_{n}^{2}$. To model the correlation of the natural channels, let $h_{AB}=\mathrm{vec}\left(H_{AB}\right)$, and define $R_{AE,AB}=E\;\left[h_{AE}h_{AB}^{H}\right]\in C^{N\times N^{2}}$, $R_{BE,AB}=E\;\left[h_{BE}h_{AB}^{H}\right]\in C^{N\times N^{2}}$, then for all i∈{1,2,…,N} and all $j\in \{1,2,\ldots ,N^{2}\}$, it holds that ${\left[R_{AE,AB}\right]}_{i,j}\neq 0$ and ${\left[R_{BE,AB}\right]}_{i,j}\neq 0$. In this case, Eve’s two-sided eavesdropping channels $h_{AE}$ and $h_{BE}$ are both correlated with the legitimate channel $H_{AB}$.

Deploying DARISA at both transmitter and receiver ends and utilizing the equivalent channel as the key source for key generation enhances the randomness of the key source in quasi-static scenarios. This randomness originates from the adjustable phase configuration of the dual-end DARISA. Furthermore, since the key source correlates with the adjustable phase at both ends, dual-end deployment resolves the issue of reduced key generation rate caused by correlation between legitimate and eavesdropping channels. The subsequent dual-end DARISA key generation agile switching scheme further leverages DARISA’s dynamic agile switching capabilities to expand the key space, thereby further enhancing key generation rate.

We next introduce the transmit–receive signal model of this system. Before each round of channel probing between Alice and Bob, the configurable phase-shift configurations at both legitimate ends are randomly updated once. Taking the case where Alice transmits as an example:

In the current channel probing slot, Alice sends a pilot $\sqrt{P}s$ to Bob. The received signal at Bob is then given by

(1) $$\begin{array}{c} y_{AB}=\sqrt{\frac{P}{N^{2}}}v_{B}^{H}H_{AB}v_{A}s+n_{B}. \end{array}$$

Similarly, the received signal at Eve is

(2) $$y_{AE}=\sqrt{\frac{P}{N}}h_{AE}^{H}v_{A}s+n_{AE}.$$

We now describe the transmit–receive signal model when DARISA exploits its agile property, taking the case of *K* agile reconfigurations as an example: DARISA performs *K* agile reconfigurations within one symbol period, i.e., the configurable phase-shift vectors of the two DARISAs are updated *K* times within a single symbol duration. The phase configuration vectors of Alice and Bob in the *k*-th reconfiguration are denoted by $v_{B,k},v_{A,k}\in C^{N\times 1},k=1,2,\ldots ,K$, and the phase configurations in different reconfigurations are assumed to be mutually independent. Taking again the case where Alice transmits as an example, after the *K*-th agile reconfiguration of the dual-ended metasurface antennas, the received signal at Bob is

(3) $$y_{AB}=\sqrt{\frac{P}{KN^{2}}}\;{\overset{ˇ}{V}}_{B}^{H}{\overset{ˇ}{H}}_{AB}{\overset{ˇ}{v}}_{A}s+n_{B}.$$

Here,

$${\overset{ˇ}{V}}_{B}=\left[\begin{array}{cccc} v_{B,1} & 0 & \ldots & 0\\ 0 & v_{B,2} & \ldots & 0\\ \vdots & \vdots & \ddots & \vdots \\ 0 & 0 & \ldots & v_{B,K} \end{array}\right]\in C^{NK\times K},\;{\overset{ˇ}{H}}_{AB}=\left[\begin{array}{cccc} H_{AB} & 0 & \ldots & 0\\ 0 & H_{AB} & \ldots & 0\\ \vdots & \vdots & \ddots & \vdots \\ 0 & 0 & \ldots & H_{AB} \end{array}\right]\in C^{NK\times NK},$$

$${\overset{ˇ}{v}}_{A}={\left[v_{A,1}^{T},v_{A,2}^{T},\ldots ,v_{A,K}^{T}\right]}^{T}\in C^{NK\times 1},\;n_{B}={\left[n_{B,1},n_{B,2},\ldots ,n_{B,K}\right]}^{T}.$$

Similarly, the received signal at Eve is

(4) $$y_{AE}=\sqrt{\frac{P}{KN}}{\overset{ˇ}{H}}_{AE}^{H}{\overset{ˇ}{v}}_{A}s+n_{AE}$$

where

$${\overset{ˇ}{H}}_{AE}=\left[\begin{array}{cccc} h_{AE} & 0 & \ldots & 0\\ 0 & h_{AE} & \ldots & 0\\ \vdots & \vdots & \ddots & \vdots \\ 0 & 0 & \ldots & h_{AE} \end{array}\right]\in C^{NK\times K},\;n_{AE}={\left[n_{AE,1},n_{AE,2},\ldots ,n_{AE,K}\right]}^{T}.$$

## 3. DARISA-Based Double-Ended Key Generation Scheme

The system model of the proposed scheme is shown in Figure 3. Key generation is performed by using the equivalent channel between Alice and Bob as the key source. The proposed scheme is described in detail as follows.

### 3.1. Channel Estimation

Before Alice and Bob perform bidirectional channel probing, the configurable phase-shift vectors at both sides are randomly updated once. First, downlink channel probing is carried out, in which Alice transmits a pilot $\sqrt{P}s$ and Bob and Eve receive it. Their received signals are given in (1) and (2), respectively. After applying least-squares channel estimation at Bob and Eve, we obtain

(5) $${\hat{h}}_{AB}=v_{B}^{H}H_{AB}v_{A}+n_{B}^{\prime },\;{\hat{h}}_{AE}=h_{AE}^{H}v_{A}+n_{AE}^{\prime }$$

where $n_{B}^{\prime }=\frac{Ns^{*}}{\sqrt{P}{\left|s\right|}^{2}}n_{B}$ and $n_{AE}^{\prime }=\frac{\sqrt{N}s^{*}}{\sqrt{P}{\left|s\right|}^{2}}n_{AE}$.

Define $\Phi_{ij}=\theta_{B,i}-\theta_{A,j}$. Since $\theta_{A,i},\theta_{B,i}∼U\left(0,2\pi \right)$, we have $\Phi_{ij}∼U\left(0,2\pi \right)$ and $\Phi_{ij}$ is independent of the channel coefficient $h_{AB,ij}$. By stacking all channel coefficients in order, we obtain $h={\left[h_{AB,11},h_{AB,12},\ldots ,h_{AB,NN}\right]}^{T}\in C^{N^{2}\times 1}$, and define the phase vector $v={\left[e^{j\Phi_{11}},e^{j\Phi_{12}},\ldots ,e^{j\Phi_{NN}}\right]}^{T}\in C^{N^{2}\times 1}$. For any (i,j), we have $h_{AB,ij}∼\mathrm{CN}\left(0,\sigma_{b}^{2}\right)$ and the entries are mutually independent, so h is a jointly complex Gaussian random vector. Hence,

$$Y=v_{B}^{H}H_{AB}v_{A}=v^{T}h.$$

**Theorem 1.**  *When the number of DARISA elements N is sufficiently large, Y follows a complex Gaussian distribution with zero mean and variance $N^{2}\sigma_{b}^{2}$, and the realizations of Y in any two channel probing slots are mutually independent.*

**Proof of Theorem 1.**  For an arbitrary channel probing, the entries of the phase vector v, i.e., $e^{j\Phi_{ij}}$, satisfy $|e^{j\Phi_{ij}}|=1$. Conditional on the phase vector v, v can be regarded as a constant, and $Y|v=v^{T}h$ is a linear combination of the jointly complex Gaussian random vector h. According to the closure property of complex Gaussian random vectors under linear transformations [26], Y∣v is still a complex Gaussian random variable, whose conditional mean and conditional variance are

$$E\left[Y|v\right]=v^{T}E\left[h\right]=0,$$

$$\mathrm{Var}\left(Y|v\right)={E[|Y|}^{2}|v]=E\;\left[v^{T}hh^{H}v^{*}\;|\;v\right]=v^{T}E\left[hh^{H}\right]v^{*}=N^{2}\sigma_{b}^{2}.$$

Since the conditional mean and variance above do not depend on the specific value of v, we have $Y|v∼\mathrm{CN}(0,N^{2}\sigma_{b}^{2})$ for any v, and thus the unconditional distribution is $Y∼\mathrm{CN}(0,N^{2}\sigma_{b}^{2})$. It can be calculated that $n_{B}^{\prime }$ follows a complex Gaussian distribution with zero mean and variance $\frac{\sigma_{n}^{2}}{P/N^{2}}$, so ${\hat{h}}_{AB}∼\mathrm{CN}(0,N^{2}\sigma_{b}^{2}+\frac{\sigma_{n}^{2}}{P/N^{2}})$.

The above derivation treats the channel as a random variable under slow fading. If we consider the extreme case where the channel varies extremely slowly or is even constant, then the value of $h_{AB,ij}$ at the current probing instant should be regarded as a deterministic constant. For the random variable $Y_{ij}=h_{AB,ij}e^{j\Phi_{ij}}$, the randomness is entirely introduced by $\Phi_{ij}$, and thus

$$E\left[Y_{ij}\right]=h_{AB,ij}\cdot E\left[e^{j\Phi_{ij}}\right]=0,$$

$$\begin{array}{cc} \mathrm{Var}(Y_{ij}) & =E\left[|Y_{ij}{|}^{2}\right]-{\left|E\left[Y_{ij}\right]\right|}^{2}=E\left[{\left|h_{AB,ij}e^{j\Phi_{ij}}\right|}^{2}\right]\\ \; & =|h_{AB,ij}{|}^{2}E\left[|e^{j\Phi_{ij}}{|}^{2}\right]={\left|h_{AB,ij}\right|}^{2}. \end{array}$$

By the generalized central limit theorem [27], $Y=\sum_{i=1}^{N}\sum_{j=1}^{N}Y_{ij}$ approximately follows a complex Gaussian distribution with mean zero and variance $\sum_{i=1}^{N}\sum_{j=1}^{N}{\left|h_{AB,ij}\right|}^{2}$. When *N* is sufficiently large, the law of large numbers implies that $\sum_{i=1}^{N}\sum_{j=1}^{N}|h_{AB,ij}{|}^{2}\approx N^{2}\sigma_{b}^{2}$. Hence we again obtain ${\hat{h}}_{AB}∼\mathrm{CN}\left(0,N^{2}\sigma_{b}^{2}+\frac{\sigma_{n}^{2}}{P/N^{2}}\right)$.

We now prove that $Y(t_{1})$ and $Y(t_{2})$ are independent in two different probing slots. In quasi-static scenarios, the channel varies slowly and we distinguish the following two cases:

1. The channel remains unchanged during the two probings.
  Denote the natural channel between Alice and Bob in the first probing slot by $H_{AB}$, so $H_{AB}\left(t_{1}\right)=H_{AB}\left(t_{2}\right)$. In this case,
  (6) $$\begin{array}{cc} \mathrm{Cov}(Y\left(t_{1}\right),Y\left(t_{2}\right)) & =E\left[v_{B}^{H}\left(t_{1}\right)H_{AB}\left(t_{1}\right)v_{A}\left(t_{1}\right)v_{A}^{H}\left(t_{2}\right)H_{AB}^{H}\left(t_{2}\right)v_{B}\left(t_{2}\right)\right]\\ \; & =E\left[\sum_{m=1}^{N}\sum_{n=1}^{N}h_{AB,mn}\left(t_{1}\right)e^{j\Phi_{mn}\left(t_{1}\right)}\cdot \sum_{p=1}^{N}\sum_{q=1}^{N}h_{AB,pq}^{*}\left(t_{1}\right)e^{-j\Phi_{pq}\left(t_{2}\right)}\right]\\ \; & \overset{\left(a\right)}{=}E\left[\sum_{m=1}^{N}\sum_{n=1}^{N}{\left|h_{AB,mn}\left(t_{1}\right)\right|}^{2}e^{j(\Phi_{mn}\left(t_{1}\right)-\Phi_{mn}\left(t_{2}\right))}\right]\\ \; & \overset{\left(b\right)}{=}0. \end{array}$$
  Here, when (m,n)≠(p,q), $h_{AB,mn}\left(t_{1}\right)$ and $h_{AB,pq}\left(t_{1}\right)$ are independent, which leads to equality (a). Since $\Phi_{mn}∼U\left(0,2\pi \right)$ and is independent of the channel coefficient, equality (b) holds.
2. The channel changes during the two probings.
  In this case,
  (7) $$\begin{array}{cc} \mathrm{Cov}(Y\left(t_{1}\right),Y\left(t_{2}\right)) & =E\left[v_{B}^{H}\left(t_{1}\right)H_{AB}\left(t_{1}\right)v_{A}\left(t_{1}\right)v_{A}^{H}\left(t_{2}\right)H_{AB}^{H}\left(t_{2}\right)v_{B}\left(t_{2}\right)\right]\\ \; & =E\left[\sum_{m=1}^{N}\sum_{n=1}^{N}h_{AB,mn}\left(t_{1}\right)e^{j\Phi_{mn}\left(t_{1}\right)}\cdot \sum_{p=1}^{N}\sum_{q=1}^{N}h_{AB,pq}^{*}\left(t_{2}\right)e^{-j\Phi_{pq}\left(t_{2}\right)}\right]\\ \; & \overset{\left(c\right)}{=}0. \end{array}$$
  For any m,n,p,q, $h_{AB,mn}\left(t_{1}\right)$ and $h_{AB,pq}\left(t_{2}\right)$ are independent, which leads to equality (c).

From the above analysis, we conclude that $Y(t_{1})$ and $Y(t_{2})$ are uncorrelated. Following [24], ${\left[Y\left(t_{1}\right),Y\left(t_{2}\right)\right]}^{T}$ can be approximated as a jointly complex Gaussian random vector, for which uncorrelatedness is equivalent to independence. Therefore, $Y(t_{1})$ and $Y(t_{2})$ obtained in different probing slots are independent.    □

In the downlink channel probing, Bob transmits a pilot $\sqrt{P}s$ to Alice, and the received signals at Alice and Eve are $y_{BA}=\sqrt{\frac{P}{N^{2}}}v_{B}^{H}H_{AB}v_{A}s+n_{A}$ and $y_{BE}=\sqrt{\frac{P}{N}}v_{B}^{H}h_{BE}s+n_{BE}$, respectively. After least-squares estimation at Alice and Eve, we obtain

(8) $${\hat{h}}_{BA}=v_{B}^{H}H_{AB}v_{A}+n_{A}^{\prime },\;{\hat{h}}_{BE}=v_{B}^{H}h_{BE}+n_{BE}^{\prime }$$

where $n_{A}^{\prime }=\frac{Ns^{*}}{\sqrt{P}{\left|s\right|}^{2}}n_{A}$ and $n_{BE}^{\prime }=\frac{\sqrt{N}s^{*}}{\sqrt{P}{\left|s\right|}^{2}}n_{BE}$. Using an analysis similar to that in Theorem 1, we obtain ${\hat{h}}_{BA}∼\mathrm{CN}\left(0,N^{2}\sigma_{b}^{2}+\frac{\sigma_{n}^{2}}{P/N^{2}}\right)$ and ${\hat{h}}_{BE}∼\mathrm{CN}\left(0,N\sigma_{n}^{2}+\frac{\sigma_{n}^{2}}{P/N}\right)$.

### 3.2. Key Generation

In the proposed scheme, ${\hat{h}}_{AB}$ and ${\hat{h}}_{BA}$ can be used as the key sources for secret key generation. Physical-layer key generation typically consists of channel probing, quantization, information reconciliation, and privacy amplification [3]. This paper focuses on the channel probing stage, i.e., the process of extracting useful information from the key sources. The remaining stages are identical to those in conventional schemes and are therefore omitted.

### 3.3. Derivation of the Key Capacity

Existing studies on DMA-assisted physical-layer key generation [23,24] have not taken into account the key leakage caused by the correlation between the legitimate channel and the eavesdropping channel, which can lead to a reduction in the final secret-key capacity. In this paper, we consider a more general scenario where Eve’s bidirectional eavesdropping channels $h_{AE}$ and $h_{BE}$ are both correlated with the legitimate channel $H_{AB}$. The following sections will show how deploying DARISA at both ends can mitigate key leakage. We derive a lower bound on the key capacity. According to [28], it can be expressed as

(9) $$\begin{array}{c} C_{s}=I\left({\hat{h}}_{AB};{\hat{h}}_{BA}\right)-I\left({\hat{h}}_{AB};{\hat{h}}_{AE},{\hat{h}}_{BE}\right). \end{array}$$

We next compute $I\left({\hat{h}}_{AB};{\hat{h}}_{AE},{\hat{h}}_{BE}\right)$ in (9).

It is shown in [29] that for a zero-mean complex Gaussian random vector $v={\left[\nu_{1},\nu_{2}\ldots \nu_{N}\right]}^{T}$ with covariance matrix $K=E(vv^{H})$, the entropy of v is given by $H\left(v\right)=\log_{2}\left({\left(\pi e\right)}^{N}\det\left(K\right)\right)$, where det(K) denotes the determinant of K. Thus,

(10) $$\begin{array}{cc} I({\hat{h}}_{AB};{\hat{h}}_{AE},{\hat{h}}_{BE}) & =H\left({\hat{h}}_{AB}\right)+H\left({\hat{h}}_{AE},{\hat{h}}_{BE}\right)-H\left({\hat{h}}_{AB},{\hat{h}}_{AE},{\hat{h}}_{BE}\right)\\ \; & =\log_{2}\frac{\mathrm{Var}\left({\hat{h}}_{AB}\right)\det\left(K_{E}\right)}{\det(K_{total})}. \end{array}$$

Here, ${\hat{h}}_{E}={\left[{\hat{h}}_{AE},{\hat{h}}_{BE}\right]}^{T}$, ${\hat{h}}_{AB,AE}={\left[{\hat{h}}_{AB},{\hat{h}}_{AE}\right]}^{T}$, $h_{total}={\left[{\hat{h}}_{AB},{\hat{h}}_{AE},{\hat{h}}_{BE}\right]}^{T}$, $K_{E}=E\left[{\hat{h}}_{E}{\hat{h}}_{E}^{H}\right]$, $K_{AB,AE}=E\left[{\hat{h}}_{AB,AE}{\hat{h}}_{AB,AE}^{H}\right]$, and $K_{total}=E\left[h_{total}h_{total}^{H}\right]$. Since the phase vectors $v_{A}$ and $v_{B}$ are independent of the channel terms, and the noise terms $n_{B}$, $n_{AE}$, and $n_{BE}$ are mutually independent and also independent of the aforementioned terms, we obtain

(11) $$\begin{array}{cc} E\;\left[{\hat{h}}_{AB}{\hat{h}}_{BE}^{*}\right]\; & =E\;\left[\left(v_{B}^{H}H_{AB}v_{A}+n_{B}^{\prime }\right){\left(v_{B}^{H}h_{BE}+n_{BE}^{\prime }\right)}^{*}\right]\\ \; & =E\;\left[\left(v_{B}^{H}H_{AB}v_{A}\right){\left(v_{B}^{H}h_{BE}\right)}^{*}\right]+E\;\left[\left(v_{B}^{H}H_{AB}v_{A}\right){\left(n_{BE}^{\prime }\right)}^{*}\right]\\ \; & \;+E\;\left[n_{B}^{\prime }{\left(v_{B}^{H}h_{BE}\right)}^{*}\right]+E\;\left[n_{B}^{\prime }{\left(n_{BE}^{\prime }\right)}^{*}\right]\\ \; & \overset{\left(d\right)}{=}E\;\left[\mathrm{tr}\;\left(v_{B}^{H}H_{AB}v_{A}h_{BE}^{H}v_{B}\right)\right]=\mathrm{tr}\;\left(E\;\left[v_{A}h_{BE}^{H}v_{B}v_{B}^{H}H_{AB}\right]\right)\\ \; & \overset{\left(e\right)}{=}\mathrm{tr}\;\left(E\left[v_{A}\right]\;E\;\left[h_{BE}^{H}v_{B}v_{B}^{H}H_{AB}\right]\right)\overset{\left(f\right)}{=}\mathrm{tr}\left(0\right)=0. \end{array}$$

Here, tr(·) denotes the trace operator. Equality (d) follows from the independence between the noise terms and the channel terms. By using the cyclic property of the trace and the independence between the channel terms and the phase vectors, we obtain equality (e). Finally, since $\theta_{A,i}∼U\left(0,2\pi \right)$, it follows that $E[v_{A}]=0$, which yields equality (f). Similarly, we have

(12) $$\begin{array}{cc} E\;\left[{\hat{h}}_{AE}{\hat{h}}_{BE}^{*}\right] & =E\;\left[\left(h_{AE}^{H}v_{A}+n_{AE}^{\prime }\right)\left(v_{B}^{H}h_{BE}+{\left(n_{BE}^{\prime }\right)}^{*}\right)\right]\\ & =E\;\left[h_{AE}^{H}v_{A}v_{B}^{H}h_{BE}\right]+E\;\left[h_{AE}^{H}v_{A}{\left(n_{BE}^{\prime }\right)}^{*}\right]+E\;\left[n_{AE}^{\prime }v_{B}^{H}h_{BE}\right]+E\;\left[n_{AE}^{\prime }{\left(n_{BE}^{\prime }\right)}^{*}\right]\\ & \overset{\left(g\right)}{=}\mathrm{tr}\;\left(E\left[v_{A}\right]\;E\left[v_{B}^{H}\right]\;E\;\left[h_{BE}h_{AE}^{H}\right]\right)=0. \end{array}$$

Equality (g) holds because the channel terms are independent of the noise terms and hence are independent of the last three terms in the expectation. Moreover, since $\theta_{A,i},\theta_{B,i}∼U\left(0,2\pi \right)$, we finally obtain $E\;\left[{\hat{h}}_{AE}{\hat{h}}_{BE}^{*}\right]=0$. From the results of $E\left[{\hat{h}}_{AB}{\hat{h}}_{BE}^{*}\right]$ and $E\left[{\hat{h}}_{AE}{\hat{h}}_{BE}^{*}\right]$, the matrix $K_{total}$ can be simplified as

$$K_{total}=\left[\begin{array}{ccc} \mathrm{Var}({\hat{h}}_{AB}) & E({\hat{h}}_{AB}{\hat{h}}_{AE}^{*}) & 0\\ E({\hat{h}}_{AE}{\hat{h}}_{AB}^{*}) & \mathrm{Var}({\hat{h}}_{AE}) & 0\\ 0 & 0 & \mathrm{Var}({\hat{h}}_{BE}) \end{array}\right]=\left[\begin{array}{cc} K_{AB,AE} & 0\\ 0^{T} & \mathrm{Var}({\hat{h}}_{BE}) \end{array}\right].$$

Therefore,

(13) $$\begin{array}{cc} I({\hat{h}}_{AB};{\hat{h}}_{AE},{\hat{h}}_{BE}) & =\log_{2}\frac{\mathrm{Var}\left({\hat{h}}_{AB}\right)\det\left(K_{E}\right)}{\det(K_{total})}=\log_{2}\frac{\mathrm{Var}\left({\hat{h}}_{AB}\right)\mathrm{Var}\left({\hat{h}}_{AE}\right)\mathrm{Var}\left({\hat{h}}_{BE}\right)}{\det\left(K_{AB,AE}\right)\mathrm{Var}\left({\hat{h}}_{BE}\right)}\\ \; & =\log_{2}\frac{\mathrm{Var}\left({\hat{h}}_{AB}\right)\mathrm{Var}\left({\hat{h}}_{AE}\right)}{\det(K_{AB,AE})}. \end{array}$$

Moreover,

(14) $$\begin{array}{cc} I({\hat{h}}_{AB};{\hat{h}}_{AE}) & =H\left({\hat{h}}_{AB}\right)+H\left({\hat{h}}_{AE}\right)-H\left({\hat{h}}_{AB},{\hat{h}}_{AE}\right)\\ \; & =\log_{2}\frac{\mathrm{Var}\left({\hat{h}}_{AB}\right)\mathrm{Var}\left({\hat{h}}_{AE}\right)}{\det(K_{AB,AE})}. \end{array}$$

Hence,

(15) $$\begin{array}{c} I\left({\hat{h}}_{AB};{\hat{h}}_{AE},{\hat{h}}_{BE}\right)=I\left({\hat{h}}_{AB};{\hat{h}}_{AE}\right). \end{array}$$

The above results indicate that, conditioned on the equivalent channel ${\hat{h}}_{AE}$, the equivalent channels ${\hat{h}}_{AB}$ and ${\hat{h}}_{BE}$ are conditionally independent. By location symmetry, it can similarly be derived that, conditioned on the equivalent channel ${\hat{h}}_{BE}$, the equivalent channels ${\hat{h}}_{AB}$ and ${\hat{h}}_{AE}$ are conditionally independent. During the evaluations of $E\left[{\hat{h}}_{AB}{\hat{h}}_{BE}^{*}\right]$ and $E\left[{\hat{h}}_{AE}{\hat{h}}_{BE}^{*}\right]$, the random phase vector $v_{A}$ guarantees that $I\left({\hat{h}}_{AB};{\hat{h}}_{AE},{\hat{h}}_{BE}\right)=I\left({\hat{h}}_{AB};{\hat{h}}_{AE}\right)$ holds. This is precisely a key feature of deploying DARISA: the antenna-side phase randomness introduced by DARISA makes the equivalent channel between the legitimate users conditionally independent of the eavesdropper’s equivalent channels. If conventional antennas were used at both ends, this property would not hold; due to the correlation between the eavesdropping and legitimate channels, potential key leakage may occur, thereby reducing the key capacity.

According to basic principles of information theory, for two zero-mean complex Gaussian random variables *X* and *Y*, their mutual information can be expressed as $I\left(X;Y\right)=-\log_{2}\left(1-{\left|\rho \right|}^{2}\right)$, where $\rho =\frac{\mathrm{Cov}(X,Y)}{\sqrt{\mathrm{Var}(X)\mathrm{Var}(Y)}}$. Here, Cov(X,Y) denotes the covariance between *X* and *Y*, and Var(X) and Var(Y) denote the variances of *X* and *Y*, respectively. Defining $\rho_{1}=\frac{\mathrm{Cov}({\hat{h}}_{AB},{\hat{h}}_{AE})}{\sqrt{\mathrm{Var}\left({\hat{h}}_{AB}\right)\mathrm{Var}\left({\hat{h}}_{AE}\right)}}$, we obtain

(16) $$\begin{array}{cc} \mathrm{Cov}\left({\hat{h}}_{AB},{\hat{h}}_{AE}\right)\; & =E\left[{\hat{h}}_{AB}{\hat{h}}_{AE}^{*}\right]=E\left[\left(v_{B}^{H}H_{AB}v_{A}+n_{B}^{\prime }\right){\left(h_{AE}^{H}v_{A}+n_{AE}^{\prime }\right)}^{*}\right]\\ \; & \overset{\left(h\right)}{=}E\left[v_{B}^{H}H_{AB}v_{A}{\left(h_{AE}^{H}v_{A}\right)}^{*}\right]\\ \; & =E\left[v_{B}^{H}H_{AB}v_{A}v_{A}^{H}h_{AE}\right]\overset{\left(i\right)}{=}E\left[v_{B}^{H}\right]E\left[H_{AB}v_{A}v_{A}^{H}h_{AE}\right]=0. \end{array}$$

Here, equality (h) follows from the independence between the channel terms and the noise terms, while equality (i) follows from the independence between the two factors in the expectation. Since $\theta_{B,i}∼U\left(0,2\pi \right)$, it follows that $\rho_{1}=0$, and hence the term $I\left({\hat{h}}_{AB};{\hat{h}}_{AE},{\hat{h}}_{BE}\right)$ in (9) is also equal to zero.

Similarly, for the term $I\left({\hat{h}}_{AB};{\hat{h}}_{BA}\right)$ in (9), we define $\rho_{2}=\frac{\mathrm{Cov}({\hat{h}}_{AB},{\hat{h}}_{BA})}{\sqrt{\mathrm{Var}\left({\hat{h}}_{AB}\right)\mathrm{Var}\left({\hat{h}}_{BA}\right)}}$. Straightforward calculations show that $\mathrm{Cov}\left({\hat{h}}_{AB},{\hat{h}}_{BA}\right)=N^{2}\sigma_{b}^{2}$ and $\mathrm{Var}\left({\hat{h}}_{AB}\right)=\mathrm{Var}\left({\hat{h}}_{BA}\right)=N^{2}\sigma_{b}^{2}+\frac{\sigma_{n}^{2}}{P/N^{2}}$. Therefore,

$$I\left({\hat{h}}_{AB};{\hat{h}}_{BA}\right)=-\log_{2}\left(1-{\left(\frac{\sigma_{b}^{2}}{\sigma_{b}^{2}+\sigma_{n}^{2}/P}\right)}^{2}\right).$$

Substituting the above results into (9) yields

(17) $$\begin{array}{cc} C_{s}\; & =I\left({\hat{h}}_{AB};{\hat{h}}_{BA}\right)-I\left({\hat{h}}_{AB};{\hat{h}}_{AE},{\hat{h}}_{BE}\right)\\ & =I\left({\hat{h}}_{AB};{\hat{h}}_{BA}\right)\\ & =-\log_{2}\left(1-{\left(\frac{\sigma_{b}^{2}}{\sigma_{b}^{2}+\sigma_{n}^{2}/P}\right)}^{2}\right)=-\log_{2}\left(1-{\left(\frac{\gamma }{1+\gamma }\right)}^{2}\right) \end{array}$$

where $\gamma =\frac{P\sigma_{b}^{2}}{\sigma_{n}^{2}}$.

In summary, the proposed scheme has the following advantages:

1. Random phases enhance the randomness of the key source and address the problem of insufficient key-source randomness under slow channel variation. By deploying DARISA at both legitimate ends and independently applying random phase configurations, the scheme introduces double-sided randomness. As shown by the analysis in Theorem 1, even under the extreme condition of a constant channel, the proposed scheme can still achieve performance close to that in time-varying environments.
2. The two-sided randomness introduced by deploying DARISA at both ends can address the reduction in the final key capacity caused by key leakage when the legitimate and eavesdropping channels are correlated. In particular, the two-sided random phases offset the impact of the intrinsic channel correlation on key extraction. The DMA-based scheme in [24] only considers deploying a metasurface antenna on one side and generating keys from the equivalent channel. As indicated by (17), due to the correlation of the underlying physical channels, one has $\mathrm{Cov}\;\left({\hat{h}}_{AB},\;{\hat{h}}_{AE}\right)\neq 0$, and therefore it is impossible to further conclude that $I\;\left({\hat{h}}_{AB};\;{\hat{h}}_{AE},\;{\hat{h}}_{BE}\right)=0$. By contrast, the proposed scheme can eliminate the leakage term induced by channel correlation, thereby providing improved security performance. Moreover, the final key capacity increases with γ; hence, for a fixed noise power, increasing the transmit power can further enhance the performance.

## 4. Dual-Ended DARISA-Based Dynamic Agile Key Generation Scheme

Dynamic agile reconfiguration is the core feature of DARISA. This property enables DARISA to adjust its phase response multiple times within a single symbol duration in a dynamic and agile manner. Based on this capability, multiple observations of different dimensions of the signal can be obtained without expanding the signal bandwidth, which provides additional degrees of freedom for performance enhancement. The dual-ended DARISA-based dynamic agile key generation scheme is an enhanced version of the dual-ended DARISA-based key generation scheme. By further exploiting the agile reconfigurability of DARISA during uplink and downlink channel probing, more observations are collected and the key generation rate can be effectively improved.

The system model of this scheme is identical to that of the dual-ended DARISA-based key generation scheme. The details are given as follows.

### 4.1. Key Generation

When the dual-ended metasurface antennas undergo the *k*-th agile reconfiguration, the configurable phase vectors at Alice and Bob are both randomly updated. First, Alice transmits a pilot $\sqrt{P}s$ to Bob, whose received signal is $y_{AB,k}=\sqrt{\frac{P}{KN^{2}}}\;v_{B,k}^{H}H_{AB}v_{A,k}s+n_{B,k}$. Then Bob sends a pilot to Alice, and the received signal at Alice is $y_{BA,k}=\sqrt{\frac{P}{KN^{2}}}\;v_{B,k}^{H}H_{AB}v_{A,k}s+n_{A,k}$. After *K* agile reconfigurations, the received signal at Bob is given in (3), while the received signal at Alice can be written as

(18) $$y_{BA}=\sqrt{\frac{P}{KN^{2}}}\;{\overset{ˇ}{V}}_{B}^{H}{\overset{ˇ}{H}}_{AB}{\overset{ˇ}{v}}_{A}s+n_{A}$$

where $n_{A}={\left[n_{A,1},n_{A,2},\ldots ,n_{A,K}\right]}^{T}$. The received signal at Eve is

(19) $$y_{BE}=\sqrt{\frac{P}{KN}}\;V_{B}^{H}h_{BE}s+n_{BE}$$

where $V_{B}=\left[v_{B,1},v_{B,2},\ldots ,v_{B,K}\right]\in C^{N\times K}$ and $n_{BE}={\left[n_{BE,1},n_{BE,2},\ldots ,n_{BE,K}\right]}^{T}$. Alice and Bob perform least-squares (LS) channel estimation, which yields

(20) $${\tilde{h}}_{AB}={\overset{ˇ}{V}}_{B}^{H}\;{\overset{ˇ}{H}}_{AB}\;{\overset{ˇ}{v}}_{A}+n_{B}^{\prime },\;{\tilde{h}}_{BA}={\overset{ˇ}{V}}_{B}^{H}\;{\overset{ˇ}{H}}_{AB}\;{\overset{ˇ}{v}}_{A}+n_{A}^{\prime }$$

where $n_{A}^{\prime }=\frac{N\sqrt{K}s^{*}}{\sqrt{P}{\left|s\right|}^{2}}{\left[n_{A,1},n_{A,2},\ldots ,n_{A,K}\right]}^{T}$ and $n_{B}^{\prime }=\frac{N\sqrt{K}s^{*}}{\sqrt{P}{\left|s\right|}^{2}}{\left[n_{B,1},n_{B,2},\ldots ,n_{B,K}\right]}^{T}$. Eve also applies LS channel estimation and obtains

(21) $${\tilde{h}}_{AE}={\overset{ˇ}{H}}_{AE}^{H}{\overset{ˇ}{v}}_{A}+n_{AE}^{\prime },\;{\tilde{h}}_{BE}={\overset{ˇ}{V}}_{B}^{H}h_{BE}+n_{BE}^{\prime }$$

where $n_{AE}^{\prime }=\frac{\sqrt{NK}s^{*}}{\sqrt{P}{\left|s\right|}^{2}}{\left[n_{AE,1},n_{AE,2},\ldots ,n_{AE,K}\right]}^{T}$ and $n_{BE}^{\prime }=\frac{\sqrt{NK}s^{*}}{\sqrt{P}{\left|s\right|}^{2}}{\left[n_{BE,1},n_{BE,2},\ldots ,n_{BE,K}\right]}^{T}$.

### 4.2. Key Capacity Analysis

The key capacity for this scheme is given by

(22) $$\begin{array}{c} C_{s}=I\left({\tilde{h}}_{AB};{\tilde{h}}_{BA}\right)-I\left({\tilde{h}}_{AB};{\tilde{h}}_{AE},{\tilde{h}}_{BE}\right). \end{array}$$

We first evaluate $I\left({\tilde{h}}_{AB};{\tilde{h}}_{AE},{\tilde{h}}_{BE}\right)$ in (22):

(23) $$\begin{array}{cc} I({\tilde{h}}_{AB};{\tilde{h}}_{AE},{\tilde{h}}_{BE})\; & =H\left({\tilde{h}}_{AB}\right)+H\left({\tilde{h}}_{AE},{\tilde{h}}_{BE}\right)-H\left({\tilde{h}}_{AB},{\tilde{h}}_{AE},{\tilde{h}}_{BE}\right)\\ \; & =\log_{2}\left(\det\left(K_{AB}\right)\right)+\log_{2}\left(\det\left(K_{E}\right)\right)-\log_{2}\left(\det\left(K_{total}\right)\right). \end{array}$$

where ${\tilde{h}}_{E}={\left[{\tilde{h}}_{AE}^{T},{\tilde{h}}_{BE}^{T}\right]}^{T}$, ${\tilde{h}}_{AB,AE}={\left[{\tilde{h}}_{AB}^{T},{\tilde{h}}_{AE}^{T}\right]}^{T}$, $h_{total}={\left[{\tilde{h}}_{AB}^{T},{\tilde{h}}_{AE}^{T},{\tilde{h}}_{BE}^{T}\right]}^{T}$, and $K_{E}=E\left[{\tilde{h}}_{E}{\tilde{h}}_{E}^{H}\right]$, $K_{AB,AE}=E\left[{\tilde{h}}_{AB,AE}{\tilde{h}}_{AB,AE}^{H}\right]$, $K_{total}=E\left[h_{total}h_{total}^{H}\right]$. Based on the scenario where Eve’s eavesdropping channels at both ends are correlated with the legitimate channel, we derive the following:

$$K_{total}=\left(\begin{array}{ccc} K_{AB} & K_{AB,AE} & 0\\ K_{AE,AB} & K_{AE} & 0\\ 0 & 0 & K_{BE} \end{array}\right).$$

Let $K_{1}=\left(\begin{array}{cc} K_{AB} & K_{AB,AE}\\ K_{AE,AB} & K_{AE} \end{array}\right)$. After some algebra, we obtain

$$I\left({\tilde{h}}_{AB};{\tilde{h}}_{AE},{\tilde{h}}_{BE}\right)=\log_{2}\left(\det\left(K_{AB}\right)\right)+\log_{2}\left(\det\left(K_{AE}\right)\right)-\log_{2}\left(\det\left(K_{1}\right)\right)=I\left({\tilde{h}}_{AB};{\tilde{h}}_{AE}\right).$$

According to [30], for two *K*-dimensional zero-mean complex Gaussian random vectors x and y whose component pairs $\left(x_{k},y_{k}\right)$ are independent and identically distributed, and satisfy $I(x_{i};y_{j})=0$ for i≠j, we have

(24) $$I\left(x;y\right)=\sum_{k=1}^{K}I\left(x_{k};y_{k}\right)=KI\left(x_{1};y_{1}\right).$$

Since the phase vectors corresponding to different agile reconfiguration rounds are mutually independent, following the same reasoning as in the proof of Theorem 1, we obtain $I({\hat{h}}_{AB}\left(i\right);{\hat{h}}_{AE}\left(j\right))=0$ for i≠j. Therefore,

(25) $$I\left({\tilde{h}}_{AB};{\tilde{h}}_{AE}\right)=\sum_{i=1}^{K}I\left({\hat{h}}_{AB}\left(i\right);{\hat{h}}_{AE}\left(i\right)\right)=K\cdot I\left({\hat{h}}_{AB}\left(1\right);{\hat{h}}_{AE}\left(1\right)\right).$$

Using the same method as in Section 3 for calculating $I({\hat{h}}_{AB};{\hat{h}}_{AE})$, and with the signal-to-noise ratio $\gamma =\frac{P\sigma_{b}^{2}}{\sigma_{n}^{2}}$, we finally obtain from (22):

(26) $$C_{s}=-K\log_{2}\left(1-{\left(\frac{\sigma_{b}^{2}}{\sigma_{b}^{2}+K\sigma_{n}^{2}/P}\right)}^{2}\right)=-K\log_{2}\left(1-{\left(\frac{\gamma }{\gamma +K}\right)}^{2}\right).$$

The proposed scheme is thus an enhanced version of the dual-ended DARISA-based key generation scheme. By exploiting the dynamic agile property of DARISA, the key space is further expanded and better performance can be achieved. From the expression above, the key capacity depends on the number of agile reconfigurations *K*. Next, we analyze how to choose *K* in low-SNR and medium-to-high-SNR regimes.

1. *Low-SNR regime.*
  When the SNR is low and a large *K* is adopted, we have $\frac{\gamma }{\gamma +K}\approx \frac{\gamma }{K}$. Then
  (27) $$\begin{array}{cc} C_{s}\; & \overset{\left(i\right)}{\approx }-K\log_{2}\;\left(1-\frac{\gamma^{2}}{K^{2}}\right)=-\frac{K}{\ln2}\;\ln\;\left(1-\frac{\gamma^{2}}{K^{2}}\right)\overset{\left(j\right)}{\approx }\frac{\gamma^{2}}{K\ln2}. \end{array}$$
  where the approximation in (j) is obtained by applying a Taylor expansion to the logarithm term. This indicates that in the low-SNR regime, if a very large *K* is used such that γ≪K, the key capacity decreases almost linearly with *K*. In practice, physical-layer key generation is usually carried out at medium or high SNR so that the key disagreement rate remains low and the subsequent reconciliation becomes easier. Therefore, we do not further focus on the low-SNR case in the simulation section.
2. *Medium-to-high-SNR regime.*
  For medium-to-high SNR, if K≪γ, we have $\frac{\gamma }{\gamma +K}\approx 1-\frac{K}{\gamma }$, and hence $1-{\left(\frac{\gamma }{\gamma +K}\right)}^{2}\approx 1-{\left(1-\frac{K}{\gamma }\right)}^{2}\approx \frac{2K}{\gamma }$. Thus,
  (28) $$C_{s}\approx -K\log_{2}\left(\frac{2K}{\gamma }\right)=K\left[\log_{2}\left(\gamma \right)-\log_{2}\left(2K\right)\right].$$
  When K≪γ, the term $\log_{2}\left(2K\right)$ varies much more slowly than $\log_{2}\left(\gamma \right)$. For a fixed SNR, the key capacity therefore grows approximately linearly with *K*, leading to improved performance. However, if *K* continues to increase until γ≪K, the capacity behavior becomes similar to that in the low-SNR case, and further increasing *K* no longer improves performance. Consequently, in the high-SNR regime, the key capacity first increases and then decreases with *K*, implying that there exists an optimal *K*. Differentiating the above expression with respect to *K* does not yield a simple closed-form solution, but numerical methods (such as the fminbnd function in MATLAB) can be used to find an approximate optimum, which is
  (29) K≈0.651γ.

Since *K* is constrained by hardware limitations, this optimal value may not be achievable in practice. In most realistic scenarios with K≪γ, increasing *K* leads to an approximately linear performance gain. Therefore, by properly exploiting the dynamic agile reconfigurability of DARISA, the key source can be extended from a random scalar to a random vector, which significantly enhances the key capacity. Moreover, the dual-ended DARISA deployment can effectively reduce the correlation between the eavesdropping and legitimate channels, thereby providing stronger security.

In summary, this scheme has the following advantages:

1. The random phase configuration enhances the randomness of the key source and addresses the lack of randomness under slowly varying channels. By deploying DARISA at both ends, independent random phases are introduced on both sides.
2. Two-end randomness resolves the issue of key leakage caused by the correlation between legitimate and eavesdropping channels, which ultimately reduces key capacity. It effectively eliminates the adverse effects arising from channel correlation.
3. Using metasurface antennas for key generation achieves key space expansion, and appropriately selecting the number of agile changes can effectively increase key capacity. DARISA’s dynamic agile characteristics enable multiple rapid and agile phase configuration changes within a single symbol period, providing more observational information.

## 5. Simulation Results and Analysis

Monte Carlo simulations are conducted to evaluate the proposed schemes. Specifically, $10^{5}$ independent trials are performed and the averaged results are reported. The mutual information is computed using the Information Theoretical Estimator (ITE) toolbox. The large-scale fading is modeled by a distance-dependent path-loss model, where the average power gain of an arbitrary link i→j is given by $\beta_{ij}=L_{0}d_{ij}^{-\alpha_{ij}}$. Here, $d_{ij}$ denotes the Euclidean distance between the nodes, $L_{0}$ is the path-loss coefficient at the reference distance (set to 1m), and $\alpha_{ij}$ is the path-loss exponent. All node coordinates are given in meters (m), so $d_{ij}$ is measured in meters, consistent with the reference distance of 1m in the path-loss model. In the simulations, $L_{0}=-30\;\mathrm{dB}$ and the receiver noise power is set to $\sigma_{n}^{2}=-95\;\mathrm{dBm}$. The path-loss exponents of the direct links are set to $\alpha_{ab}=\alpha_{ae}=3$. To ensure a fair comparison, the user locations in the RIS-based scheme remain unchanged, and path loss is still taken into account. In addition, the reflecting-surface-related links in [17] use $\alpha_{r}=2.5$. Alice, Bob, and Eve are located at (0,0), (65,0), and (65,2), respectively. The RIS is deployed at (0,1). The number of elements on one side of DARISA is set to N=16, and the number of RIS elements is set to 32. For small-scale fading, Rayleigh fading with spatial correlation is considered, and the correlated channels are generated according to the Jakes model. The simulations not only demonstrate the performance of the proposed DARISA-based schemes, but also compare them with the classical RIS-based random phase-tuning scheme in [17] and the DMA-based scheme that generates keys from the equivalent channel in [24].

**Figure 4:**
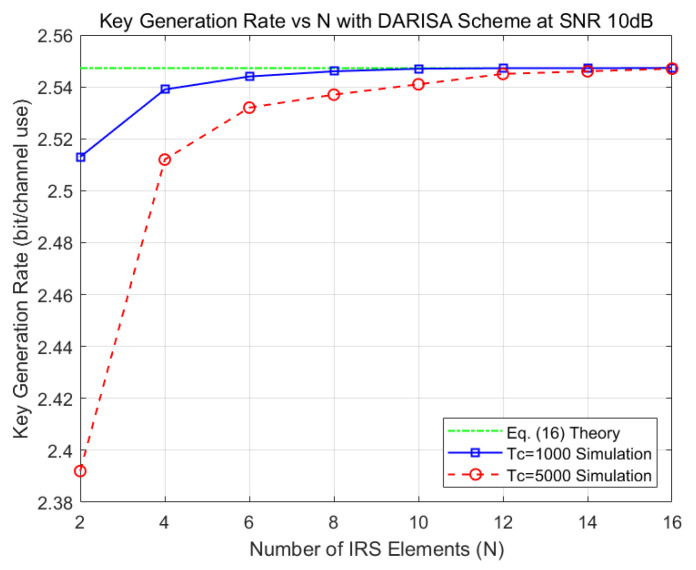
Key generation rate of the DARISA scheme versus *N* at SNR = 10 dB.

Figure 4 shows the key generation rate of the DARISA-based scheme versus the number of elements *N* for a fixed SNR of 10 dB. In the figure, $T_{c}=1000$ corresponds to a slowly varying channel, while $T_{c}=5000$ represents the extreme case of an almost static channel. It can be observed that even in the extreme case of a static channel, increasing *N* allows the simulated key generation rate to approach the theoretical value in (16), and only around ten elements are required to reach this limit. These results verify the correctness of the analysis in Section 3 and demonstrate the feasibility of the proposed scheme.

**Figure 5:**
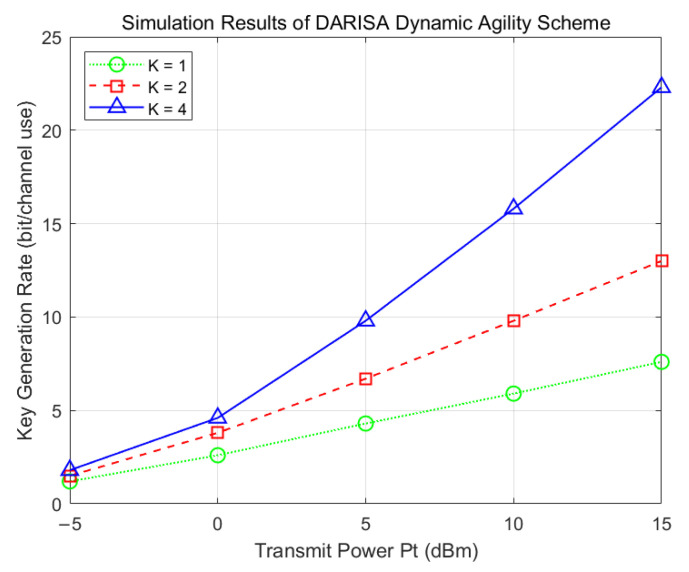
Simulation results of the dual-ended DARISA-based dynamic agile key generation scheme.

Figure 5 presents the simulation results for the dual-ended DARISA deployment with dynamic agile operation. As the number of agile reconfigurations increases, the key generation rate improves accordingly. This is because exploiting the agile property of DARISA effectively transforms the key source from a random scalar into a random vector, thereby enriching the available randomness.

**Figure 6:**
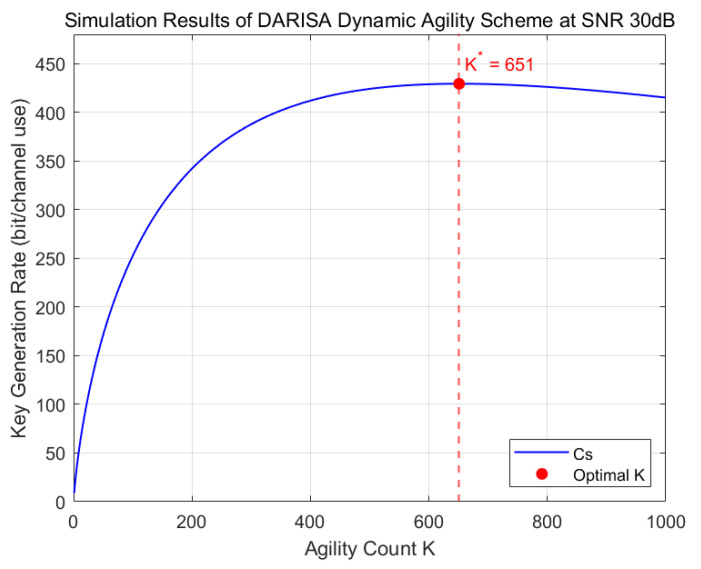
Simulation results of the DARISA dynamic agile scheme at SNR = 30 dB.

Figure 6 illustrates the impact of the number of agile reconfigurations on the key generation performance at an SNR of 30 dB. The key generation rate first increases and then decreases as the number of reconfigurations grows, and the optimal value of the reconfiguration number matches well with the analytical result in (29).

**Figure 7:**
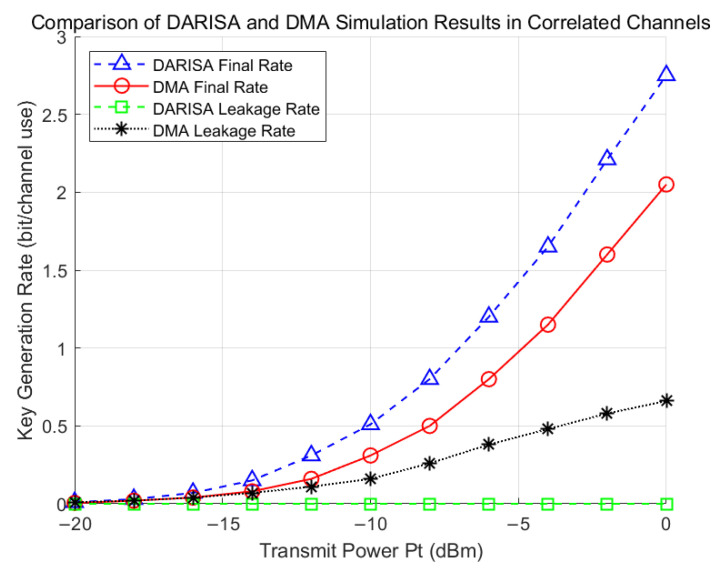
Comparison between the DARISA scheme and the DMA scheme under correlated channels.

Figure 7 compares the DARISA-based scheme in [24] and the DMA-based scheme under correlated channels. When a single DMA is deployed at one side and the eavesdropping channel is correlated with the legitimate channel (for example, when Eve is close to Bob), the information leakage rate of the DMA-based scheme increases with the transmit power and degrades the final key generation rate. In contrast, the DARISA-based scheme introduces double-sided random phases, which effectively mitigate the impact of channel correlation between the eavesdropper and the legitimate users and thus improve the secrecy performance.

**Figure 8:**
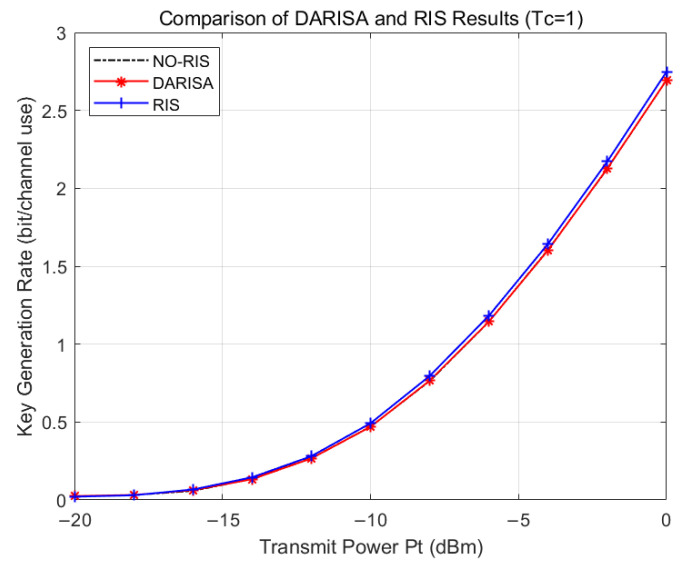
Simulation comparison of DARISA and RIS schemes for $T_{c}=1$.

Figure 8 shows the comparison between the DARISA-based and RIS-based schemes for $T_{c}=1$, which corresponds to a fast-varying channel. In this case, the RIS-aided key generation scheme achieves better performance, since the RIS introduces additional propagation paths when the channel varies rapidly, leading to a higher key generation rate. For the NO-RIS channel-based key generation scheme, its performance at this point aligns with that of the DARISA scheme. This is because the performance upper bound of the DARISA scheme when not employing dynamic agility features corresponds to the channel-time-varying channel-based key generation result. However, the agility feature of DARISA can introduce additional degrees of freedom to achieve superior performance, as demonstrated in Figure 5.

**Figure 9:**
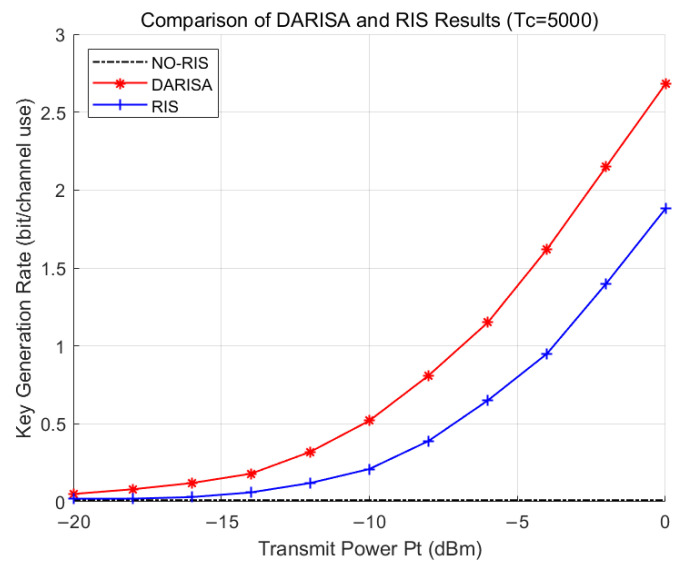
Simulation comparison of DARISA and RIS schemes for $T_{c}=5000$.

In Figure 9, when $T_{c}=5000$, the channel corresponds to a quasi-static (static) case. In this situation, generating secret keys solely based on the channel may result in an excessively low key generation rate, or even make key generation infeasible. Compared with RIS-assisted physical-layer key generation schemes, the DARISA scheme can achieve better performance in such slowly varying or even invariant channel scenarios.

## 6. Conclusions

In this paper, we introduce DARISA into physical-layer key generation to reshape the wireless environment and propose a dual-ended DARISA-based key generation scheme together with an enhanced dynamic agile version. The dual-ended scheme improves key randomness in quasi-static environments through independently configurable phases at both legitimate ends, while the resulting double-sided randomness eliminates the performance loss caused by correlation between the legitimate and eavesdropping channels. Building on this, the dynamic agile scheme further exploits DARISA’s time-varying capability, increasing the amount of observations during uplink and downlink channel probing, expanding the key space, and enhancing the key generation rate. We also compare the performance differences between the DARISA and DMA schemes and the RIS scheme. Compared to the DMA scheme, this approach eliminates the adverse effects caused by natural channel correlations through dual-end randomization. Since RIS can only reshape the wireless environment on the same side and under slow-varying channel conditions, DARISA demonstrates superior performance and broader application scenarios.

## Data Availability

No new data were created or analyzed in this study. Data sharing is not applicable to this article.

## References

[1] Sasi T., Habibi Lashkari A., Amin R., Mantar H. (2024). A comprehensive survey on IoT attacks: Taxonomy, detection mechanisms and challenges. J. Inf. Intell..

[2] Alshamaseen T., Althunibat S., Qaraqe M., Pervaiz H., Lestas M. (2021). Secure key distribution for IoT networks based on physical layer security. Proceedings of the 2021 IEEE 26th International Workshop on Computer Aided Modeling and Design of Communication Links and Networks (CAMAD).

[3] Li G., Yu J., Hu A. (2020). Research on physical-layer security based on device and channel characteristics. J. Cryptologic Res..

[4] Wang Y., Xu Y., Liu J., Liu Y., Wang S., Han W., Wang Y. (2024). A practical scheme for enhancing consistency and independence in Wi-Fi networks benefit from physical layer wireless key generation. Phys. Commun..

[5] Du Y., Liu J., Wang Q., Liu J., Wang Y. (2024). Secure and controllable secret key generation through CSI obfuscation matrix encapsulation. IEEE Trans. Mob. Comput..

[6] Han B., Li Y., Wang X., Li H., Huang J. (2023). FLoRa: Sequential fuzzy extractor based physical layer key generation for LPWAN. Future Gener. Comput. Syst..

[7] Yang Z., Xu Y., Sun P., Wu C. (2024). BlueKey: Exploiting Bluetooth Low Energy for enhanced physical-layer key generation. Proceedings of the IEEE Conference on Computer Communications (INFOCOM).

[8] Xia E., Hu B.J., Shen Q. (2024). A survey of physical layer secret key generation enhanced by intelligent reflecting surface. Electronics.

[9] Jangsher S., Al-Dweik A., Iraqi Y., Pandey A., Giacalone J.-P. (2023). Group secret key generation using physical layer security for UAV swarm communications. IEEE Trans. Aerosp. Electron. Syst..

[10] Tang Z., Zhang M., Kim S. (2024). Efficient physical-layer secret key generation in satellite communication system. Proceedings of the International Conference on ICT Convergence (ICTC).

[11] Huang J. (2021). Research on secret key generation based on wireless channel characteristics in body area network. Telecommun. Sci..

[12] Zhao H., Guo E., Lian Z., Zhang J., Guo D., Xiang Y. (2024). A review and implementation of physical layer channel key generation in the Internet of Things. J. Inf. Secur. Appl..

[13] Keshavarzi M., Zayyani H., Kuhestani A., Mokari N., Chen D. (2024). A new practical physical layer secret key generation in the presence of an untrusted relay. Phys. Commun..

[14] Aldaghri N., Mahdavifar H. (2020). Physical layer secret key generation in static environments. IEEE Trans. Inf. Forensics Secur..

[15] Sandell M. (2023). Secret key generation with multiantenna relays. IEEE Access.

[16] Hasan S.R., Sabuj S.R., Hamamura M., Hossain M.A. (2024). A comprehensive review on reconfigurable intelligent surface for 6G communications: Overview, deployment, control mechanism, application, challenges, and opportunities. Wirel. Pers. Commun..

[17] Hu X., Jin L., Huang K., Sun X., Zhou Y., Qu J. (2021). Intelligent reflecting surface-assisted secret key generation with discrete phase shifts in static environment. IEEE Wirel. Commun. Lett..

[18] Lu T., Chen L., Zhang J., Chen C., Hu A. (2023). Joint precoding and phase shift design in reconfigurable intelligent surfaces-assisted secret key generation. IEEE Trans. Inf. Forensics Secur..

[19] Ajayi I., Medjahdi Y., Zayani R., Mroueh L., Kaddour F.Z. (2022). PAPR-Aware Artificial Noise for Secure Massive MIMO Downlink. IEEE Access.

[20] Xu N., Nan G., Tao X. (2023). Passive eavesdropping can significantly slow down RIS-assisted secret key generation. Proceedings of the 2023 IEEE Global Communications Conference (GLOBECOM).

[21] Wan Z., Chu Z., Mi D., Xiao P., Zhu Z. (2024). STAR-RIS-assisted physical-layer key generation. IEEE Trans. Veh. Technol..

[22] Bai J., Wang H.-M., Jin L. (2026). Dynamic agile reconfigurable intelligent surface antenna (DARISA) MIMO: DoF analysis and effective DoF optimization. IEEE Trans. Wirel. Commun..

[23] Hao Y.N., Zhong Z., Sun X.L., Jin L. (2022). DMA-based key generation method for IoT scenario. J. Commun..

[24] Yang J., Ji X.S., Huang K.Z., Zhao J.L., Guan X.R. (2022). Secret key generation scheme based on RIS antenna for static environments. Sci. Sin. Inform..

[25] Sleasman T.A., Imani M.F., Diebold A.V., Boyarsky M., Trofatter K.P., Smith D.R. (2021). Dynamic Metasurface Aperture for Computational Microwave Imaging. IEEE Trans. Antennas Propag..

[26] Jing Y., Yu X. (2020). Lecture Series on Math Fundamentals for MIMO Communications, Topic 1: Complex Random Vector and Circularly Symmetric Complex Gaussian Matrix. https://www.ece.ualberta.ca/~yindi/MathBackground/Topic-1-ComplexGaussian-2020-01-17.pdf.

[27] Li G., Hu A., Zhang J., Xiao B. (2017). Security analysis of a novel artificial randomness approach for fast key generation. Proceedings of the 2017 IEEE Global Communications Conference (GLOBECOM).

[28] Zhang J., He B., Duong T.Q., Woods R. (2017). On the key generation from correlated wireless channels. IEEE Commun. Lett..

[29] Wallace J.W. (2009). Secure physical layer key generation schemes: Performance and information theoretic limits. Proceedings of the 2009 IEEE International Conference on Communications (ICC).

[30] Tse D., Viswanath P. (2005). Fundamentals of Wireless Communication.

